# Fatty acid-induced CD36 expression via O-GlcNAcylation drives gastric cancer metastasis

**DOI:** 10.7150/thno.34024

**Published:** 2019-07-09

**Authors:** Mingzuo Jiang, Nan Wu, Bing Xu, Yi Chu, Xiaowei Li, Song Su, Di Chen, Wenjiao Li, Yanting Shi, Xiaoliang Gao, Haohao Zhang, Zhao Zhang, Wei Du, Yongzhan Nie, Jie Liang, Daiming Fan

**Affiliations:** 1 State key Laboratory of Cancer Biology, National Clinical Research Center for Digestive Diseases and Xijing Hospital of Digestive Diseases, Air Force Military Medical University, 710032, Xi'an, China.; 2 Department of Gastroenterology, Second Affiliated Hospital of Xi'an Jiaotong University, 710032, Xi'an, China; 3 Lab of Tissue Engineering, Faculty of Life Science, Northwest University,710032, Xi'an, China; 4 Department of Gastroenterology, Navy General Hospital, 100048, Beijing, China.; 5 The School of Basic Medicine, Air Force Military Medical University, 710032, Xi'an, China.

**Keywords:** fatty acid, gastric cancer, CD36, O-GlcNAcylation, metastasis

## Abstract

Metastasis is the primary cause of death in patients with advanced cancer. Recently, a high-fat diet was shown to specifically promote the metastatic potential of specific cancer cells in a CD36-dependent manner. However, the molecular basis of the fatty acid (FA)-induced upregulation of CD36 has remained unclear.

**Methods**: RT-qPCR, FACS analysis, immunoblotting and immunohistochemistry, as well as retrieving TCGA database, were carried out to quantitate CD36 expression in gastric cancer (GC) tissues and cell lines. Transwell assay and xenografts were used to assess cell metastasis abilities* in vitro* and *in vivo* after indicated treatment. Luciferase reporter assay was carried out to evaluate the changes in signaling pathways when O-GlcNAcylation level was increased in GC cells and *in vitro* O-GlcNAcylation assay was utilized for wild and mutant types of CD36 protein to explore the potential O-GlcNAcylation sites.

**Results**: High CD36 expression is a predictor of poor survival and promotes metastasis of GC cells and the use of neutralizing antibodies to block CD36 inhibits GC metastasis in mice. FA or a HFD promotes the metastatic potential of GC cells by upregulating CD36 via increasing the O-GlcNAcylation level. Increased O-GlcNAcylation levels promote the transcription of CD36 by activating the NF-κB pathway and also increase its FA uptake activity by directly modifying CD36 at S468 and T470.

**Conclusion**: FA-induced hyper-O-GlcNAcylation promotes the transcription and function of CD36 by activating the NF-κB pathway and directly modifying CD36 at S468 and T470, which drives GC metastasis.

## Introduction

Metastasis has become the most fearsome aspect of cancer because most cancer-related deaths are caused by this process 1, 2. Despite the substantial decrease in the incidence and mortality associated with gastric cancer (GC) in most parts of the world, GC remains one of the most common and fatal malignancies worldwide due to metastasis 3-5. Considerable basic and translational research has provided insights into the mechanisms of GC metastasis and has yielded significant clues regarding the crucial molecular events during the progression of GC 6-8. However, this process remains one of the most complex and enigmatic aspects of GC development.

The reprogramming of metabolic pathways is a compelling feature of cells that transform from a normal to cancerous state, a process that initiates and propagates damage that leads to cancer cell proliferation and metastasis 9, 10. Notable among these dysregulated metabolic pathways are changes in lipid metabolism, which have been posited to play a major role in cancer cell metastasis 11. In particular, upregulation of the fatty acid (FA) receptor CD36 has been identified as an important mediator in the initiation of metastasis and is closely related to clinical outcomes or metastatic progression in many types of malignancies 12. Mice fed a high-fat diet (HFD) have a higher metastatic potential of oral squamous cell carcinomas (OSCCs), accompanied by an increase in the percentage of CD36^+^ cells. Consistently, the exposure of cultured OSCC cells to palmitic acid (PA), a dietary FA recognized by CD36, also significantly increases the percentage of CD36^+^ cells and specifically promotes the metastatic potential of GC in a CD36-dependent manner 12.However, the molecular basis of the FA-induced upregulation of CD36 has remained unclear.

O-GlcNAcylation, which is catalyzed by a single enzyme termed O-linked N-acetylglucosamine transferase (OGT), plays an important role as a nutrient sensor and is correlated with glucose and lipid metabolism 13. The disruption of O-GlcNAc homeostasis and alteration of the intracellular protein level of OGT is correlated with altered metabolism in cancer cells 14-16. OGT also participates in transcriptional regulation by modulating transcription factors 17-19. Studies have shown that a HFD can augment O-GlcNAcylation levels 20 and that high levels of O-GlcNAcylation induce PA production in liver cancer cells 14. In addition, O-GlcNAcylation was also observed to increase plasma membrane levels of FAT/CD36 during cardiac metabolism 21, indicating that O-GlcNAcylation and CD36 are functionally related. However, despite these important functions, the potential effects of CD36 on GC metastasis remain unknown. More importantly, less evidence is available regarding whether O-GlcNAcylation takes part in GC metastasis, particularly by regulating CD36 expression and function.

In this study, we evaluated the significance of CD36 in GC metastasis. In particular, we characterized the functional mechanisms of O-GlcNAcylation in metastatic GC by investigating CD36 expression and function, and our results provide new insights into the potential treatment of metastatic GC in humans.

## Materials and Methods

**Cell culture:** The human GC cell lines SGC 7901, SGC 7901-NM, SGC 7901-M, MKN-28, BGC823, AGS, HGC-27, MGC-803 and MKN-45 were cultured in basic DMEM (Gibco, Grand Island, NY, USA) supplemented with 10% fetal bovine serum (Bioind, Kibbutz Beit Haemek, Israel) and 1% penicillin-streptomycin (Gibco). All cells were cultured at 37°C with 5% (vol/vol) CO_2_ and were tested for mycoplasma contamination. All cell lines except SGC-7901 were authenticated by short tandem repeat DNA profiling.

**OGT or CD36 stable knockout cells:** Stable OGT or CD36 knockout cells were established via a CRISPR/Cas9-sgRNA system. Puromycin-resistant lentiviral vectors carrying CRISPR/Cas9 and the indicated OGT-targeted (OGT KO-sgRNA1, OGT KO-sgRNA2 and OGT KO-sgRNA3) or CD36-targeted sgRNA (CD36 KO-sgRNA1, CD36 KO-sgRNA2 and CD36 KO-sgRNA3) were transfected into SGC7901 or MKN-45 cells and then selected for two weeks using puromycin to obtain stable transfected cell lines. sgRNA sequences for OGT: sg-1, TGGCTTCTTCCAAGCGACCC; sg-2, GCTCAAAGCCCTGGGTCGCT; sg-3, CACCCTTGACCCAAACTTTC; sgRNA sequences for CD36: sg-1, ACCTTTATATGTGTCGATTA; sg-2, GCCATAATCGACACATATAA; sg-3, TGATAGTGAAGGTTCGAAGA; The knockout of endogenous OGT or CD36 were confirmed by Western Blotting assay (Figure S6) and we chose to use the combined transfection of CD36 KO-sgRNA1, 2 and 3 for the next experiments in order to get the best knockout efficiency.

**Nile red staining:** Cells were fixed in 4% paraformaldehyde for 10 min and washed with PBS twice. Then, the cells were incubated with 20 μg/ml Nile red (Sigma-Aldrich, 72485, St. Louis, USA) in PBS for 30 min in the dark at room temperature. Then, the cells were washed with PBS twice and incubated for 15 min with DAPI (Sigma-Aldrich, D9542, St. Louis, USA) in the dark at room temperature. After washing with PBS twice, the cells were mounted on coverslips using an antifade solution (Chemicon®, S7114, Darmstadt, Germany).

***In vitro* O-GlcNAcylation assay:** *In vitro* O-GlcNAcylation of CD36-FL (ORIGENE, TP710013, Rockville, USA) or CD36-ES (Sino Biological, 10752-H08H, Beijing, China) was performed in 100 μL assay volumes containing 1 μg of OGT (R&D, 8446-GT, Minneapolis, USA) in reaction buffer (50 mM Tris-HCl, 1 mM DTT, and 12.5 mM MgCl_2_, pH 7.5), and 2 mM UDP-GlcNAc (Calbiochem®, 670107, San Diego, USA). The reactions were incubated for 3 h at 37°C and then heated for 5 min at 95°C with 25 μL of 5× SDS-PAGE loading buffer (Beyotime Biotechnology, P0015, Shanghai, China).

**Luciferase reporter assay:** When cells reached 60% confluence in 24-well plates, a firefly luciferase reporter gene construct (0.1 μg), miRNA construct (0.4 μg), and a Renilla luciferase construct (0.02 μg) were cotransfected into the cells using X-tremeGENE HP (Roche, 6366244001, Basel, Switzerland). Subsequently, 48 h after transfection, luciferase activity was measured using a Dual-Luciferase Reporter Assay System (Promega, E1910, Madison, USA) according to the manufacturer's instructions. To elucidate which signal transduction pathways were activated by O-GlcNAcylation, a Cignal Finder Signal Transduction 45-Pathway Reporter Array (QIAGEN, CCA-901, 336821, Dusseldorf, Germany) was used according to the manufacturer's instructions.

**Immunoprecipitation (IP) and Co-IP:** IP and co-IP were performed using a Pierce® Co-Immunoprecipitation kit (Thermo scientific, 26149, Waltham, USA) according to the manufacturer's instructions.

**Fatty acid uptake assay:** A FA uptake assay was performed using a FA uptake assay kit (BioVision, K408-100, Milpitas, USA) according to the manufacturer's instructions. Briefly, 7 × 10^4^ cells/well were seeded into a black-walled 96-well culture plate and incubated at 37°C with 5% (vol/vol) CO_2_ overnight. Then, the medium was replaced with 100 μL of serum-free, phenol red-free medium and incubated at 37°C with 5% (vol/vol) CO_2_ for 2 h. Following serum starvation, 100 μL of prewarmed 2× uptake reaction mix was added to all of the wells. The fluorescence (Ex/Em = 488/523) of all the wells was measured at the indicated times.

**Immunohistochemical (IHC) staining:** Monoclonal antibodies against O-GlcNAc (Abcam, Ab2739, Cambridge, UK) and CD36 (R&D, MAB19554, Minneapolis, USA) were used for IHC analyses, with tissue staining performed as previously published 22. On each slide, both the IHC staining score of positive cells and the intensity of the positive cells were calculated using the SDiquantitative scoring method. The immunostaining intensity was evaluated as previously described 23.

**Virus:** Virus packaging was performed in HEK 293T cells by cotransfection with lentiviral vectors with the packaging plasmid pHelper 1.0 vector (GeneChem Co., Ltd., Shanghai, China) and the envelope plasmid pHelper 2.0 vector (GeneChem Co., Ltd., Shanghai, China) using Lipofectamine 2000 (Invitrogen, Karlsruhe, Germany).

**Mice:** Six-week-old male BALB/C nude mice were used for all experiments. For the lung metastasis assay, mice (n = 10/group) were randomly administered target cells through the tail vein (2 × 10^6^ cells in 100 µL of PBS). The mice were anesthetized and sacrificed 8 weeks after injection, and histological assessments of the lungs were performed by hematoxylin-eosin (H&E) staining. HFD experiments were performed by feeding mice a 60/Fat Rodent Diet (OpenSource Diets®, D12492, New Brunswick, USA) for 1 week before being inoculated with the tumor cells. A normal diet (OpenSource Diets®, D12450J, New Brunswick, USA) was used for the control groups. Mice were anesthetized and sacrificed 8 weeks after inoculation, and histological assessments of the lungs were performed by H&E staining. To treat mice in vivo with neutralizing anti-CD36 antibodies, the mice were weekly injected intraperitoneally with 100 µL PBS containing 20 μg of anti-CD36 neutralizing monoclonal JC63.1 (CAYMAN, CAY-10009893-500, Ann Arbor, USA) or 20 μg of the corresponding IgA (Mouse IgA, kappa [S107] - Isotype Control, Abcam, ab37322, Cambridge, UK) at the beginning of the experiment. Mice were anesthetized and sacrificed 8 weeks after inoculation, and histological assessments of the lungs were performed by H&E staining. All animal procedures were performed according to the guidelines approved by the Animal Care and Use Committee of the Air Force Military Medical University, which are in accordance with NIH guidelines.

**Statistical analysis:** We used Prism 5 and SPSS 18.0 software for statistical analyses, and all values are presented as the means ± SD unless otherwise indicated.

## Results

### Upregulation of CD36 predicts poor survival and promotes cell metastasis of GC

**Figure 1:**
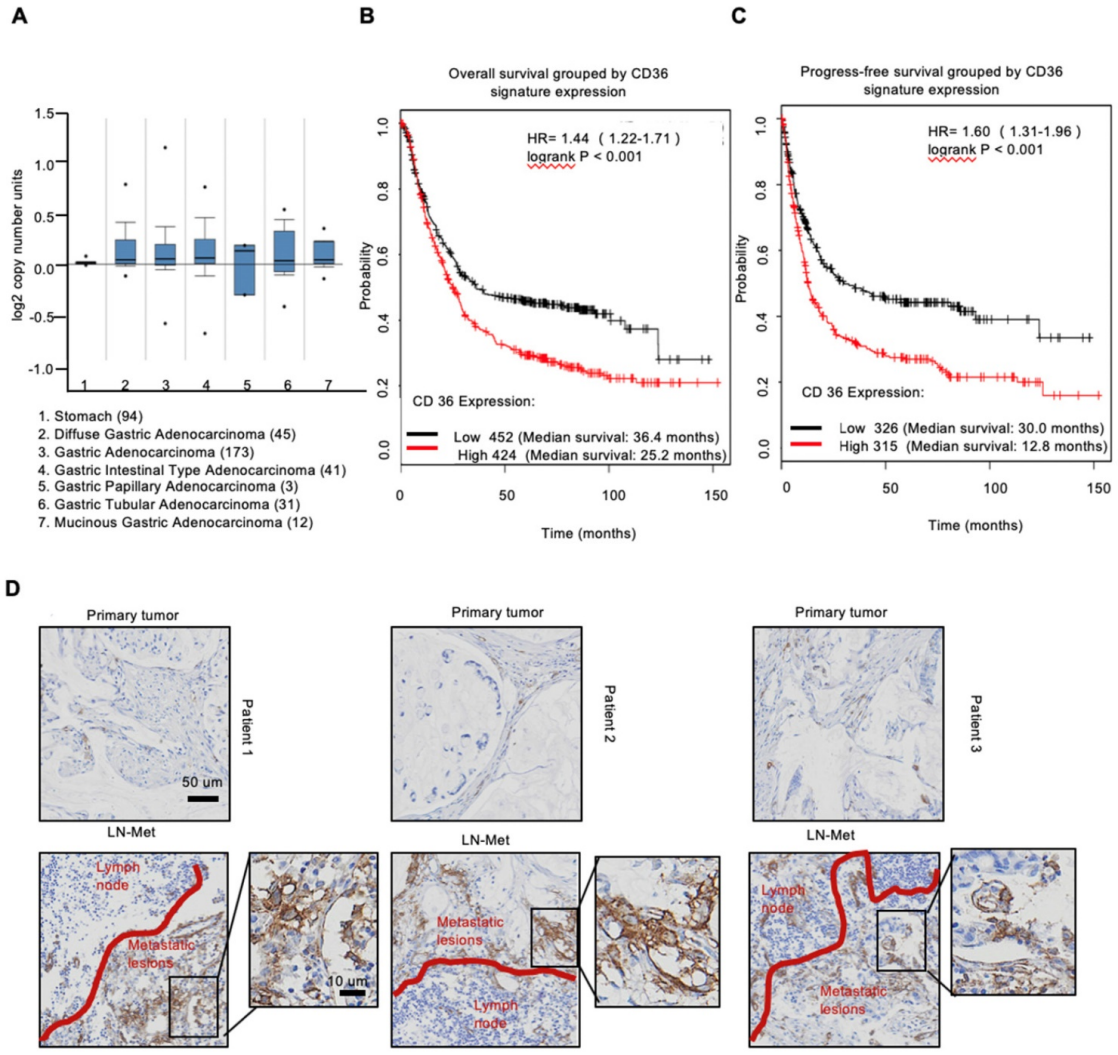
Upregulation of CD36 predicts poor survival in GC. (A) CD36 gene copy number in 305 GC tumor samples and 94 normal controls from TCGA data. The data were analyzed by ONCOMINE. (B,C) Kaplan-Meier curve depicting the OS of 876 GC patients (B) and the progression-free survival of 641 GC patients (C). The data were analyzed by a Kaplan-Meier plotter (http://kmplot.com/analysis/). (D) Representative images of IHC staining of CD36 in primary tumor tissues and positive metastatic lymph nodes of three GC patients.

**Figure 2:**
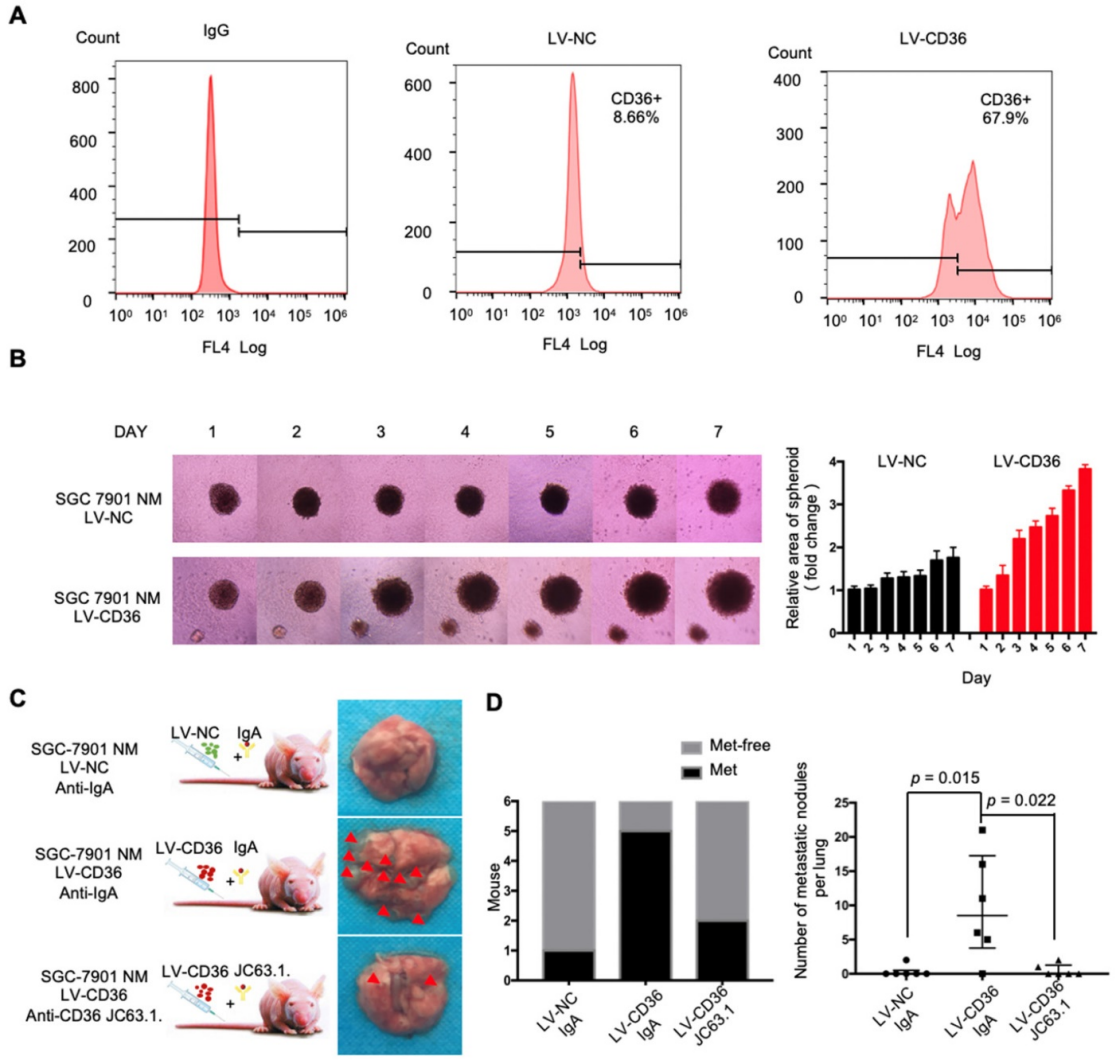
Upregulation of CD36 in GC cell promotes cell metastasis. (A) Flow cytometry analysis showing the expression of CD36 in SGC 7901-NM cells transfected with a lentiviral vector encoding CD36 or negative control vector. K isotype IgG was used as a negative control. (B)3D spheroid BME cell invasion assay of SGC 7901-NM cells transfected with lentiviral vector encoding CD36 (LV-CD36) or with negative control vector (LV-NC). Photographs of all the spheroids in each well every 24 h for 7 days using a 4× objective. Quantitative analysis of the surface area of all spheroids. Normalized areas for all the spheroids are presented relative to the area on the first day. All of the areas were calculated three times using ImageJ, and the values represent the means ± SD. (C) The indicated cells were injected into nude mice (n = 6 for each group) via the tail vein along with weekly intraperitoneal injections of 20 μg of the anti-CD36 neutralizing monoclonal antibody JC63.1 or 20 μg of the corresponding IgA. Animals were sacrificed at 8 weeks after the injections. Photos of representative lung tissue samples in each group are shown. (D) Left: The histogram shows the proportion of mice with lung metastasis in each group. “Met” is short for “metastasis,” and “Met-free” indicates “metastasis-free.” Right: Mann Whitney test was used to evaluate the number of metastatic nodes in the lungs of mice from each group.

To investigate the expression of CD36 in human gastric carcinomas, we searched The Cancer Genome Atlas (TCGA) database and noted that the CD36 gene copy number was significantly elevated in numerous types of GCs compared with that observed in normal gastric tissues (Figure 1A). We next analyzed publicly available data and observed that CD36 expression was strongly correlated with overall survival (Figure 1B) and progression-free survival (Figure 1C) in patients with GC. Importantly, significantly more CD36-positive cancer cells were observed to be present in lymph node metastatic foci than in primary tumors (Figure 1D). Moreover, we overexpressed CD36 via lentivirus infection in SGC 7901-NM, a GC cell line with low metastatic potential 24 (Figure 2A), and evaluated the effect of CD36 on metastatic capability using a three-dimensional (3D) spheroid basal membrane extract (BME) cell invasion assay (Figure 2B) and a transwell assay (Figure S1A). Interestingly, cancer cell spheroids with higher CD36 levels showed stronger migration and invasion capabilities. In addition, we also knocked out CD36 using the CRISPR/Cas9-sgRNA system in MKN-45, AGS and SGC 7901-M cells, GC cell lines with high metastatic potential 24. Transwell assays showed that the knockout of CD36 significantly reduced GC cell migration and invasion capabilities (Figure S1B-D). Furthermore, the xenograft model also showed that the number of metastatic nodules in the lung was dramatically increased when CD36 was overexpressed in SGC 7901-NM cells (Figure 2C and 2D). Correspondingly, treating mice with a weekly tail vein injection of the neutralizing antibody JC63.1 nearly completely inhibited metastasis initiation (Figure 2C and 2D). Taken together, these results strongly indicate that upregulation of CD36 is a predictor of poor survival and promotes metastasis of GC cells.

### High fat diets induced CD36 expression and promoted metastasis in mice

**Figure 3:**
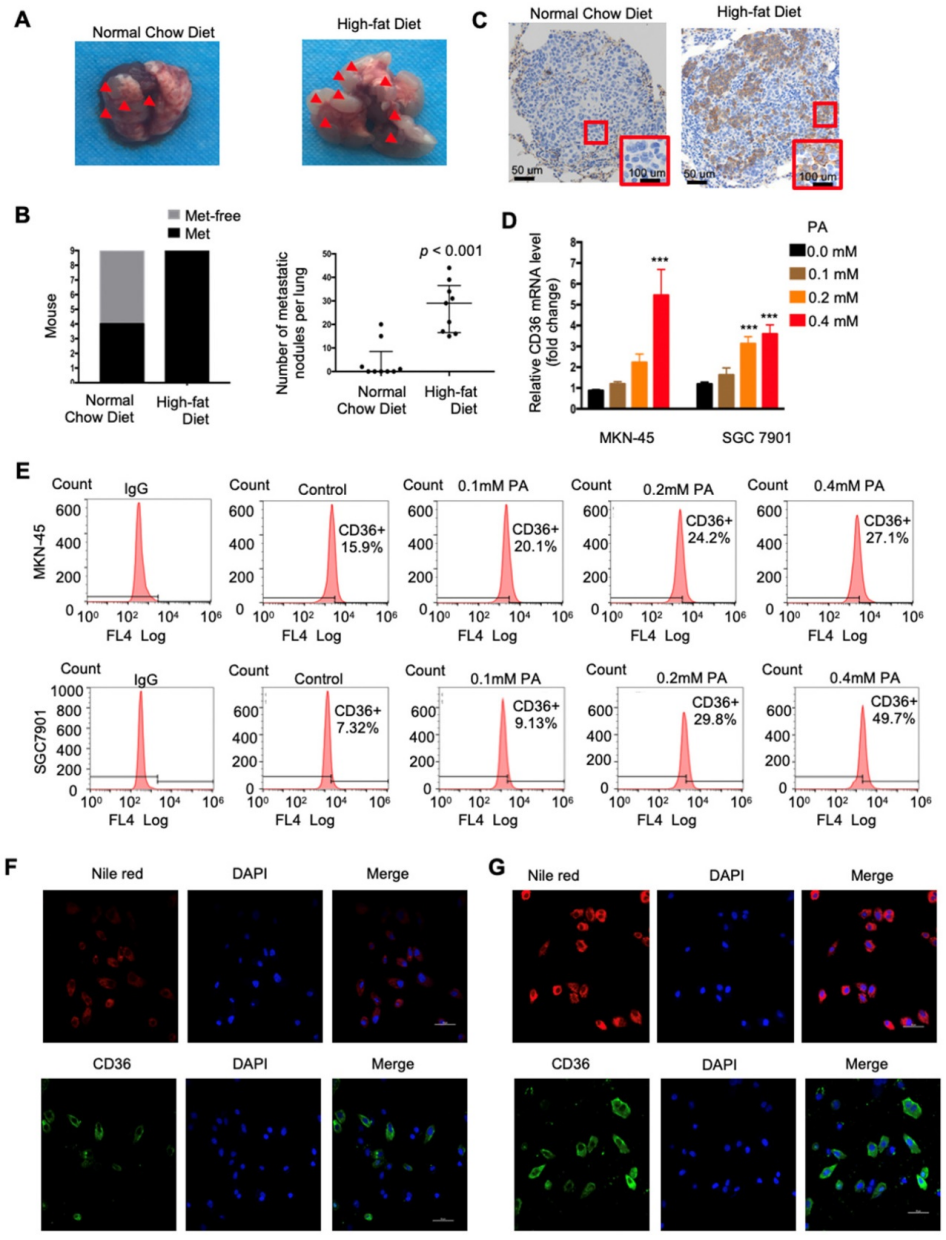
High fat diets induced CD36 expression and promoted metastasis in mice. (A) SGC 7901 cells were injected into the tail vein of nude mice (n = 9 for each group) fed either a HFD or a normal chow diet. Photos of representative lung tissue samples in each group are shown. (B) Left: The histogram shows the proportion of mice with lung metastasis in each group. “Met” is short for “metastasis,” and “Met-free” represents “metastasis-free.” Right: Mann Whitney test was used to evaluate the number of metastatic nodes in the lungs of each group. (C) Representative CD36 staining of the metastatic nodes in mouse lungs from the HFD or normal chow diet groups. (D) CD36 mRNA levels in MKN-45 or SGC 7901 cells were assessed by real-time PCR after treatment with the indicated concentration of PA or control solvent for 24 h. The values shown are expressed as the means ± SD of three independent experiments. (E) Flow cytometry analysis of CD36 levels in MKN-45 or SGC 7901 cells after treatment with the indicated concentration of PA or control solvent for 24 h. K isotype IgG was used as a negative control. (F, G) Immunofluorescence staining showed the FA and CD36 levels in SGC 7901 cells without (F) or with (G) 0.4 µM PA treatment for 24 h. FAs were stained by Nile red.

CD36 mediates the uptake and intracellular transport of long-chain FAs (LCFAs) and is sensitive to the concentration of FAs in numerous types of cells 25-27. Moreover, obesity and a HFD are well documented to be closely and positively related to the metastasis and poor prognosis of multiple tumors, including GC 28. However, the relationship between dietary lipids and CD36 in GC is not clear. Therefore, we explored whether dietary lipids promote GC metastasis by inducing CD36 expression. In the xenograft model, we observed that nude mice fed a HFD developed more and larger metastatic nodules in the lungs (Figure 3A and 3B). In addition, the results of an immunohistochemical analysis showed that the increased metastatic potential of SGC 7901 cells in HFD-fed mice correlated with an increase in the percentage of CD36^+^ cells in lung metastatic lesions (Figure 3C), suggesting that CD36 expression may also be sensitive to the concentration of FAs in GC. To further investigate the relationship between dietary lipids and CD36 in GC, we exposed cultured GC cell lines (SGC 7901 and MKN-45) to palmitic acid (PA), a dietary FA recognized by CD36, for 24 h. As expected, both the mRNA and protein levels of CD36 were robustly increased after PA treatment (Figure 3D and 3E). Moreover, immunofluorescence analysis also confirmed that the expression of CD36 in the membrane was significantly increased after 24 h of PA treatment (Figure 3F and 3G). These results show that high-fat diets promote metastasis in mice, perhaps by inducing the expression of CD36.

### PA treatment promoted metastasis and induced CD36 expression through activating the hexosamine biosynthetic pathway (HBP)

**Figure 4:**
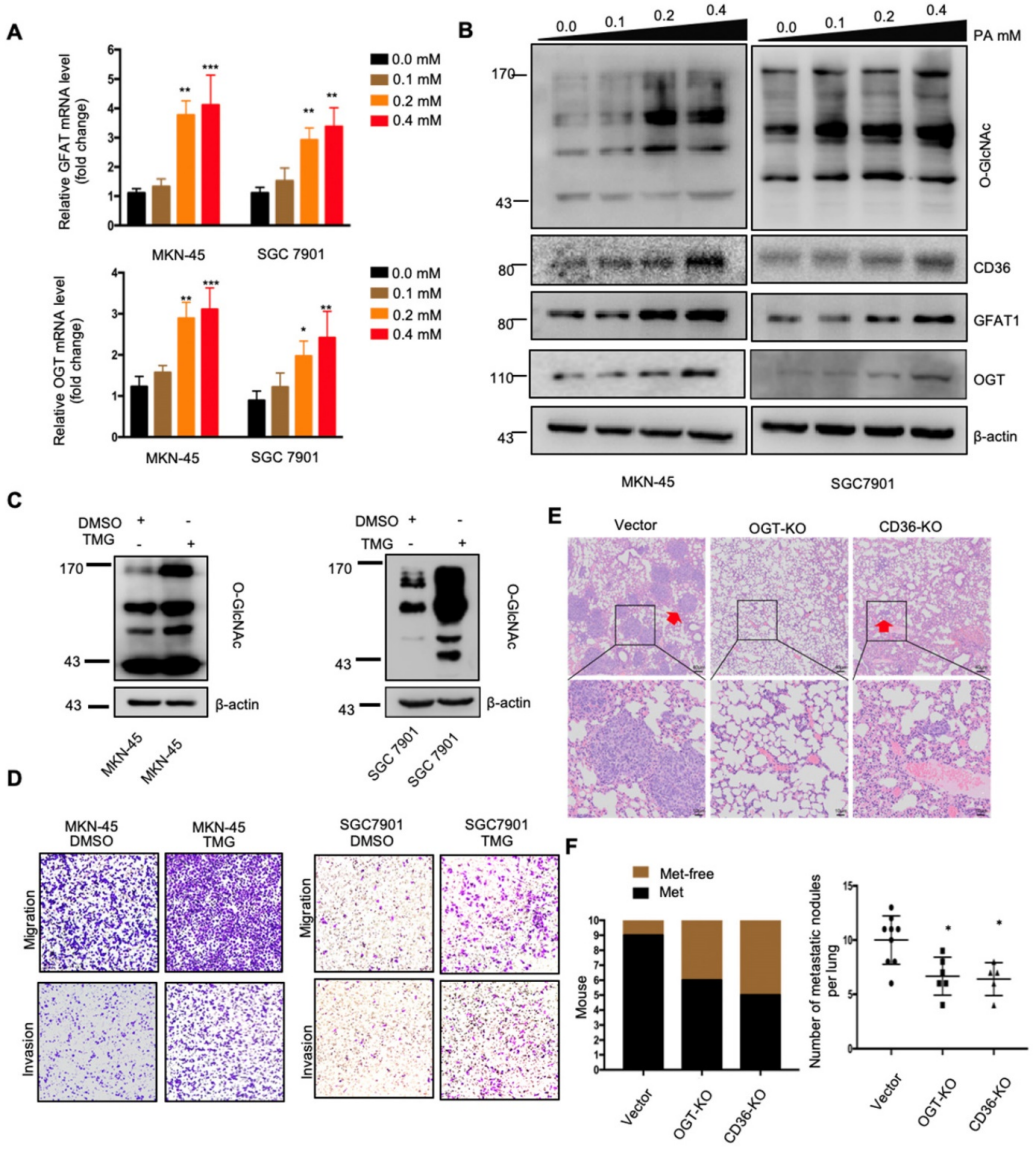
PA treatment promoted metastasis and induced CD36 expression through activating the HBP. (A) GFAT and OGT mRNA levels in MKN-45 or SGC 7901 cells were assessed by real-time PCR after treatment with the indicated concentration of PA or control solvent for 3 h. The values shown are expressed as the means ± SD of three independent experiments. (B) GFAT, OGT and CD36 levels in MKN-45 or SGC 7901 cells were assessed by western blotting after treatment with the indicated concentration of PA or control solvent for 3 h. (C) The levels of O-GlcNAcylation in MKN-45 or SGC 7901 cells were assessed by western blotting after treatment with 10 μM TMG or isometric DMSO for 24 h. β-actin was used as a loading control. (D) Transwell migration and invasion assay of MKN-45 and SGC 7901 cells after10 μM TMG or isometric DMSO treatment for 24 h. (E) Indicated cells were injected into nude mice (n = 10 for each group) via the tail vein and mice were fed with HFD. Animals were sacrificed at 6 weeks after the injections. Representative HE staining of the metastatic nodes in mouse lungs from each group. (F) Left: The histogram shows the proportion of mice with lung metastasis in each group. “Met” is short for “metastasis,” and “Met-free” represents “metastasis-free.” Right: Mann Whitney test was used to evaluate the number of metastatic nodes in the lungs of each group. * represents Mann Whitney test *p* < 0.05.

The above results indicated that dietary lipid-induced CD36 expression plays a crucial role in promoting GC metastasis. However, how dietary lipids induce CD36 overexpression and then promote metastasis in GC remains unclear. As a key nutrient sensor, the hexosamine biosynthetic pathway (HBP) has been implicated in lipid metabolism and is closely associated with multiple signal transduction pathways 15, 29-31. Moreover, the results of our previous study showed that O-GlcNAcylation is elevated in GC tissues and is associated with the proliferation and metastasis of GC 22. Therefore, we hypothesized that dietary lipids induce CD36 expression and promote the metastasis of GC by activating the HBP. To test our hypothesis, we first investigated the effect of PA on GFAT and OGT expression, two key enzymes associated with O-GlcNAc modification. The mRNA and protein levels of GFAT and OGT, as well as the CD36 and total O-GlcNAcylation levels, were significantly increased in cells when increasing concentrations of PA were administered (Figure 4A and 4B). In addition, we increased the O-GlcNAcylation level by applying thiamet-G (TMG) for 24 h and observed extremely elevated CD36 protein levels (Figure S2A). Notably, small interfering RNA (siRNA)-mediated depletion of OGT significantly blocked the increase in CD36 in SGC 7901 cells, which was mediated by treatment with 0.4 mM PA for 24 h (Figure S2B).

Subsequently, we further evaluated whether increased O-GlcNAcylation enhanced the metastatic capability of GC. When O-GlcNAcylation was upregulated by treating cells with the O-GlcNAcase inhibitor TMG (Figure 4C), the transwell assays showed that the migration and invasion capacities of MKN-45 and SGC 7901 cells were markedly increased (Figure 4D). In addition, TMG treatment also increased the invasion capacities of other GC cell lines, including BGC 823, HGC27, MGC 803, MKN-28 and AGS cells (Figure S2C). Furthermore, a 3D spheroid BME cell invasion assay using SGC 7901-M and SGC 7901-NM cells also showed the same results, with O-GlcNAcylation promoting the invasion of GC cells (Figure S2D and S2E). Importantly, the levels of O-GlcNAcylation were investigated by immunohistochemical (IHC) staining of 187 clinical tissues from GC patients, consisting of 53 normal adjacent tissues, 68 primary GC tissues and 66 metastatic GC tissues, which were assigned to three groups (negative, weak and strong) on the basis of IHC scores (Figure S3A and S3B). For individual GC patients, the O-GlcNAcylation levels in GC tissues were higher than those observed in normal adjacent tissues and were highest in metastatic GC tissues (Figure S3C). In addition, we observed that after 24 h of 0.4 µM PA treatment, the activity of several classical signal transduction pathways in the SGC 7901 cells changed significantly (Figure S4A and S4B). Cluster analysis revealed that knocking out OGT or CD36 largely reversed the PA-induced changes (Figure S4C). Moreover, knockout of OGT or CD36 partly blocked the metastasis induced by HFD in mice (Figure 4E and 4F). The results showed that dietary lipids promote metastasis and induce CD36 expression by activating the HBP.

### O-GlcNAcylation promoted CD36 transcription by activating the NF-κB pathway

**Figure 5:**
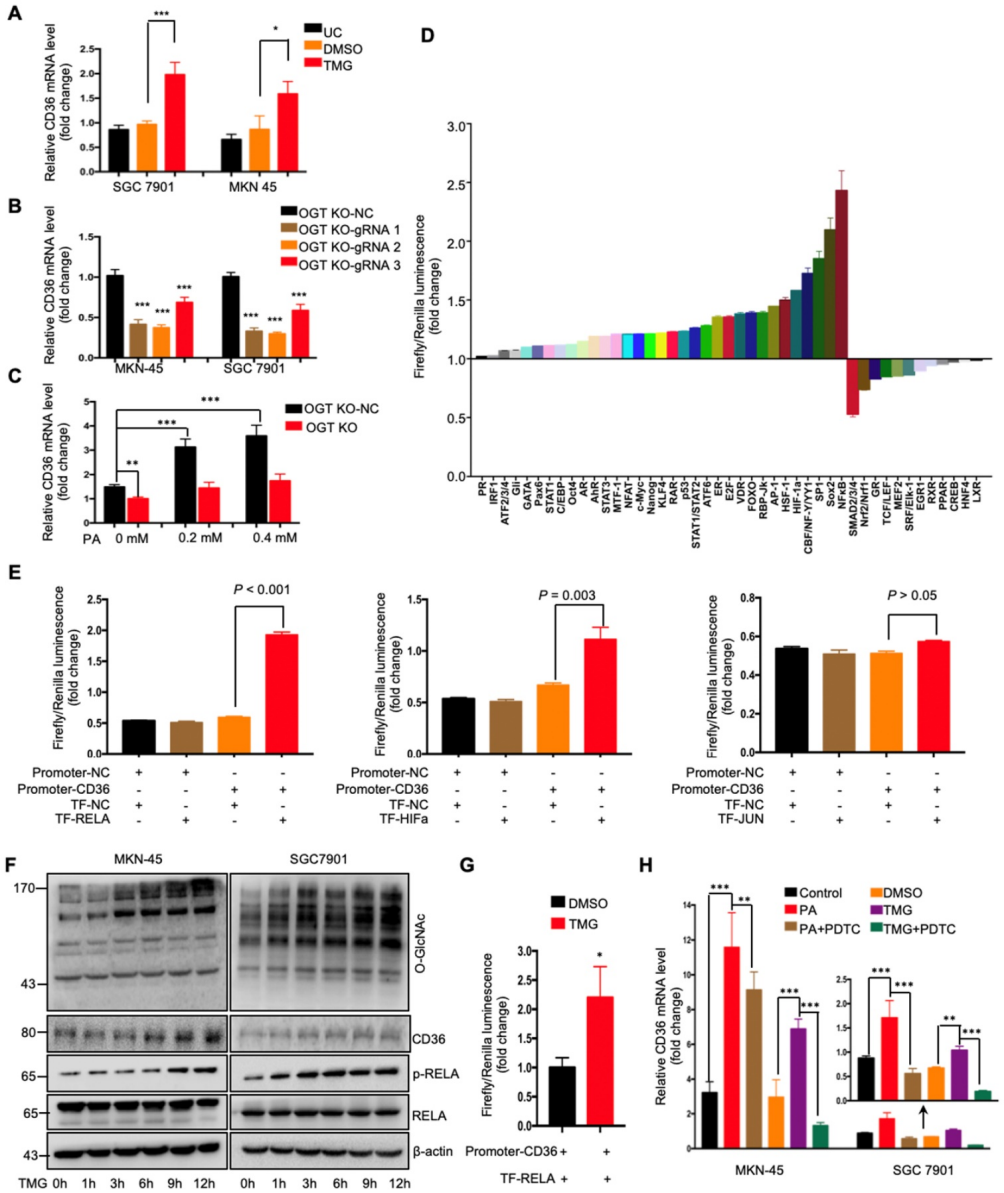
O-GlcNAcylation promoted CD36 transcription via activating the NF-κB pathway. (A) CD36 mRNA levels in MKN-45 or SGC 7901 cells were assessed by real-time PCR after a 24-hour treatment with TMG (10 μM) or isometric DMSO. (B) CD36 mRNA levels were assessed in MKN-45 or SGC 7901 OGT-knockout cells by real-time PCR. (C) CD36 mRNA levels in SGC 7901 cells with or without OGT knockout after treatment with the indicated concentration of PA for 24 h. (D) A luciferase reporter assay showed changes in the activity of 45 signal transduction pathways in SGC 7901 cells after a 24-hour treatment with TMG (10 μM). (E) A luciferase reporter assay showed the regulation of CD36 transcription by the indicated transcription factors in HEK 293T cells. (F) The levels of O-GlcNAcylation, RELA, phosphorylated RELA and CD36 in MKN-45 or SGC 7901 cells were assessed by western blotting after treatment with 10 μM TMG or isometric DMSO for the indicated times. β-actin was used as a loading control. (G) A luciferase reporter assay showed the regulation of CD36 transcription by NF-κB after treatment with 10 μM TMG or isometric DMSO for 12 h. (H) CD36 mRNA levels in MKN-45 or SGC 7901 cells were assessed by real-time PCR after the indicated treatment. The values shown are expressed as the means ± SD of three independent experiments. Control: control solvent treatment for 24 h; PA: treatment with 0.4 μM of PA for 24 h; PA+PDTC: treatment with 0.4 μM of PA for 24 h and pretreatment with 50 µmol of pyrrolidine dithiocarbamate for 4 h; DMSO: isometric DMSO treatment for 24 h; TMG: treatment with 10 μM of TMG for 24 h; TMG+PDTC: treatment with 10 μM of TMG for 24 h and pretreatment with 50 µmol of pyrrolidine dithiocarbamate for 4 h.

To further investigate how O-GlcNAcylation promotes CD36 expression, CD36 mRNA levels were assessed in MKN-45 and SGC 7901 cell lines with or without TMG pretreatment. We observed that the levels of CD36 mRNA in both cell lines increased after TMG treatment (Figure 5A), indicating that O-GlcNAcylation promotes CD36 transcription. In addition, knockout of OGT also reduced the level of CD36 mRNA in the MKN-45 and SGC 7901 cell lines (Figure 5B). Moreover, knockout of OGT in SGC 7901 cells partly blocked the PA treatment-mediated elevation of CD36 (Figure 5C). To elucidate which signal transduction pathways are activated by O-GlcNAcylation to promote the transcription of CD36, we performed a dual-luciferase reporter assay and observed that several O-GlcNAcylation-activated pathways (Figure 5D). We further observed that within these pathways, NF-κB, HIF-α and AP-1 could bind to the CD36 promoter region. Subsequently, a luciferase reporter assay was performed, the results of which showed that the luciferase activities of the reporter constructs increased most significantly when RELA constructs, rather than HIF-α or JUN constructs, were cotransfected with the CD36 reporter in HEK 293T cells (Figure 5E). Indeed, we observed that the levels of p-RELA and CD36 increased with the increase in O-GlcNAcylation in both SGC 7901 and AGS cells after treatment with TMG (10 μM) at different times (0, 1, 3, 6 h, 9 or 12 h) (Figure 5F). Furthermore, the luciferase reporter assay results also showed that TMG treatment promoted NF-κB-mediated CD36 transcription (Figure 5G). Moreover, the treatment of MKN-45 or SGC 7901 cells with an NF-κB inhibitor, pyrrolidine dithiocarbamic acid (PDTC), partly blocked the PA or TMG treatment-mediated induction of CD36 transcription (Figure 5H). Taken together, the results of these analyses revealed that O-GlcNAcylation promotes CD36 transcription by activating the NF-κB pathway.

### O-GlcNAcylation of CD36 enhances its FA uptake activity

**Figure 6:**
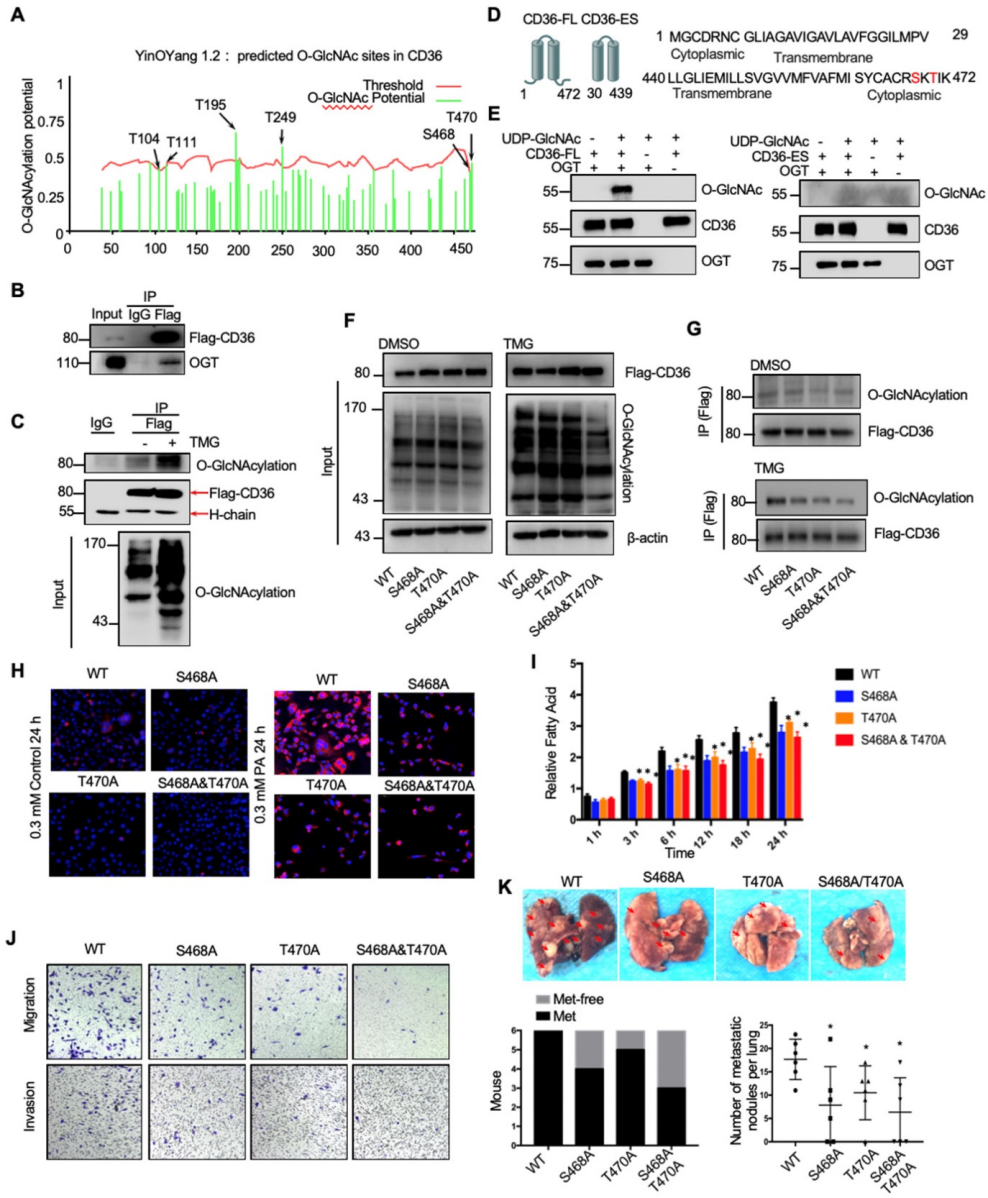
O-GlcNAcylation modification of CD36 in S468 and T470 enhances its FA uptake activity. (A) The O-GlcNAc sites of CD36 predicted by the YinOYang 1.2 server (www.cbs.dtu.dk/services/YinOYang) are shown with a black arrowhead at the top. The green vertical lines show the potential O-GlcNAc-modified Ser/Thr residues, and the red horizontal wavy line indicates the threshold for modification potential. (B) Total lysates from SGC 7901 cells expressing FLAG-tagged CD36 (FLAG-CD36) were subjected to IP with FLAG Ab, followed by western blotting using the indicated antibodies (Abs). (C) Total lysates from SGC 7901 cells expressing FLAG-tagged CD36 (FLAG-CD36) with or without TMG were subjected to IP with IgG or FLAG, followed by western blotting using O-GlcNAcylation or FLAG antibodies. (D) Schematic model of the recombinant CD36 full length (CD36-FL) protein and CD36 extracellular segment (CD36-ES). (E) O-GlcNAcylation assays using recombinant human CD36-FL or CD36-ES were performed in the presence or absence of UDP-GlcNAc or recombinant human OGT. Reaction mixtures were immunoblotted with antibodies against O-GlcNAc (RL2), CD36, and OGT. (F, G) Lentiviral vectors encoding FLAG-tagged wild-type (WT) CD36, CD36 with an alanine substitution at S468 (S468A), CD36 with an alanine substitution at T470 (T470A) or CD36 with alanine substitutions at both S468 and T470 (S468A/T470A) were transfected into SGC 7901 cells after knocking out endogenous CD36 expression. Then, the cells were treated with 10 μM TMG or isometric DMSO for 12 h before cell lysis, and then the O-GlcNAcylation of CD36 was detected. (H) Representative Nile red staining of SGC 7901 cells transfected with WT, S468A, T470A or S468A &T470A CD36 lentiviral vectors after treatment with 0.3 μM of PA or isometric control solvent for 24 h. (I) Fluorescence of SGC 7901 cells transfected with the indicated lentiviral vector. Fluorescence was measured at the indicated times after the addition of uptake reaction mix. Data are expressed as the mean RFU ± SD of three independent plates, with each data point representing the mean of quadruplicate wells. * represents Student's t test *p* < 0.05. (J) Transwell assay of SGC 7901 cells transfected with the indicated lentiviral vector after 0.4 μM PA treatment for 24 h. (K) Indicated SGC 7901 cells were injected into the tail vein of nude mice (n = 6 for each group) fed either a HFD or a normal chow diet. Photos of representative lung tissue samples in each group are shown. Left: The histogram shows the proportion of mice with lung metastasis in each group. “Met” is short for “metastasis,” and “Met-free” represents “metastasis-free.” Right: Mann Whitney test was used to evaluate the number of metastatic nodes in the lungs of each group.

Because the functions of a wide panel of oncogenic factors can be affected by their O-GlcNAcylation 15, we next explored whether CD36 can be directly modified by O-GlcNAcylation. Using the YinOYang 1.2 Server (www.cbs.dtu.dk/services/YinOYang), we predicted 6 amino acids in CD36 that may be directly modified by O-GlcNAc (Figure 6A). The results of a coimmunoprecipitation (co-IP) assay showed that exogenous FLAG-tagged CD36 was directly modified by O-GlcNAc and coprecipitated with endogenous OGT in SGC 7901 cells (Figure 6B and 6C). Furthermore, the results of an in vitro O-GlcNAcylation assay indicated that the full-length recombinant CD36 (CD36-FL) protein could be O-GlcNAcylated by recombinant OGT, whereas the extracellular segment of CD36 (CD36-ES) could not (Figure 6D and 6E). O-GlcNAcylation occurs only at serine (S) or threonine (T) residues, which are only present within the carboxyl terminus (C-terminus) of the intracellular segment of CD36 (Figure 6D). Among these residues, S468 and T470 are potential O-GlcNAcylation sites predicted by the YinOYang 1.2 Server. To determine whether S468 and T470 are major residues of O-GlcNAcylation, we exogenously expressed FLAG-tagged wild-type (WT) and CD36 mutant proteins with alanine substitutions at S468 (S468A) and T470 (T470A) alone or in combination (S468A/T470A) in SGC 7901 cells after knocking out endogenous CD36 expression. Subsequently, the cells were treated with or without TMG before cell lysis, and the O-GlcNAcylation of CD36 was assessed. The results indicated that mutating S468 or T470 to alanine notably reduced CD36 O-GlcNAcylation in SGC 7901 cells (Figure 6F and 6G). Moreover, the TMG treatment markedly enhanced the O-GlcNAcylation of WT but not mutated CD36, suggesting that the O-GlcNAcylation at S468 or T470 is highly dynamic.

To investigate the role of O-GlcNAcylation on CD36 FA uptake activity, the WT, S468A, T470A, and S468A/T470A SGC 7901 cell lines were treated with 0.3 µM PA or control solvent for 24 h, which was followed by Nile red staining to assess the FA level in the cytoplasm. Despite the FA level being increased after PA treatment in all cell lines, the FA level in the WT CD36 cell line was notably higher than that observed in the S468A, T470A, and S468A/T470A cell lines, suggesting that O-GlcNAcylation at S468 and T470 is a positive regulator of CD36 FA uptake activity (Figure 6H). Indeed, upon further examination of the uptake rate of FA in the four cell lines, the rate of FA uptake in the WT group was faster than that observed in the S468A, T470A, and S468A/T470A groups (Figure 6I). In addition, the results of a Transwell assay showed that the migration and invasion abilities of SGC 7901 (Figure 6J), MKN-45 (Figure S5A) and AGS cells (Figure S5B) decreased after 0.4 µM PA treatment for 24 h when the S468 or T470 residues of CD36 were mutated, indicating that O-GlcNAc modification of S468 or T470 is crucial for the CD36 promotion of GC metastasis. We also observed that CD36 knockout can partly block OGT overexpression (Figure S5C) and 10 µM TMG treatment (Figure S5D) mediated the invasion of SGC 7901 and MKN 45 cells. Furthermore, the expression of CD36 by plasmid transfection could rescue the invasion ability of CD36-knockout cancer cells, but the expression of CD36 with S468A, T470A or S468A/T470A mutations could not (Figure S5C and S5D). In addition, in the xenograft model of the SGC 7901 (Figure 6K) or MKN-45 cell lines (Figure S5E and S5F), we observed that the WT CD36 groups developed more metastatic nodules in the lungs than the S468A, T470A, and S468A/T470A groups when the mice were fed a HFD. Thus, the results showed that O-GlcNAcylation of CD36 at S468 and T470 enhances cellular FA uptake activity and is critical but not necessary for GC invasion.

### Association of CD36 and OGT expression with clinicopathological parameters and prognosis of GC patients

**Figure 7:**
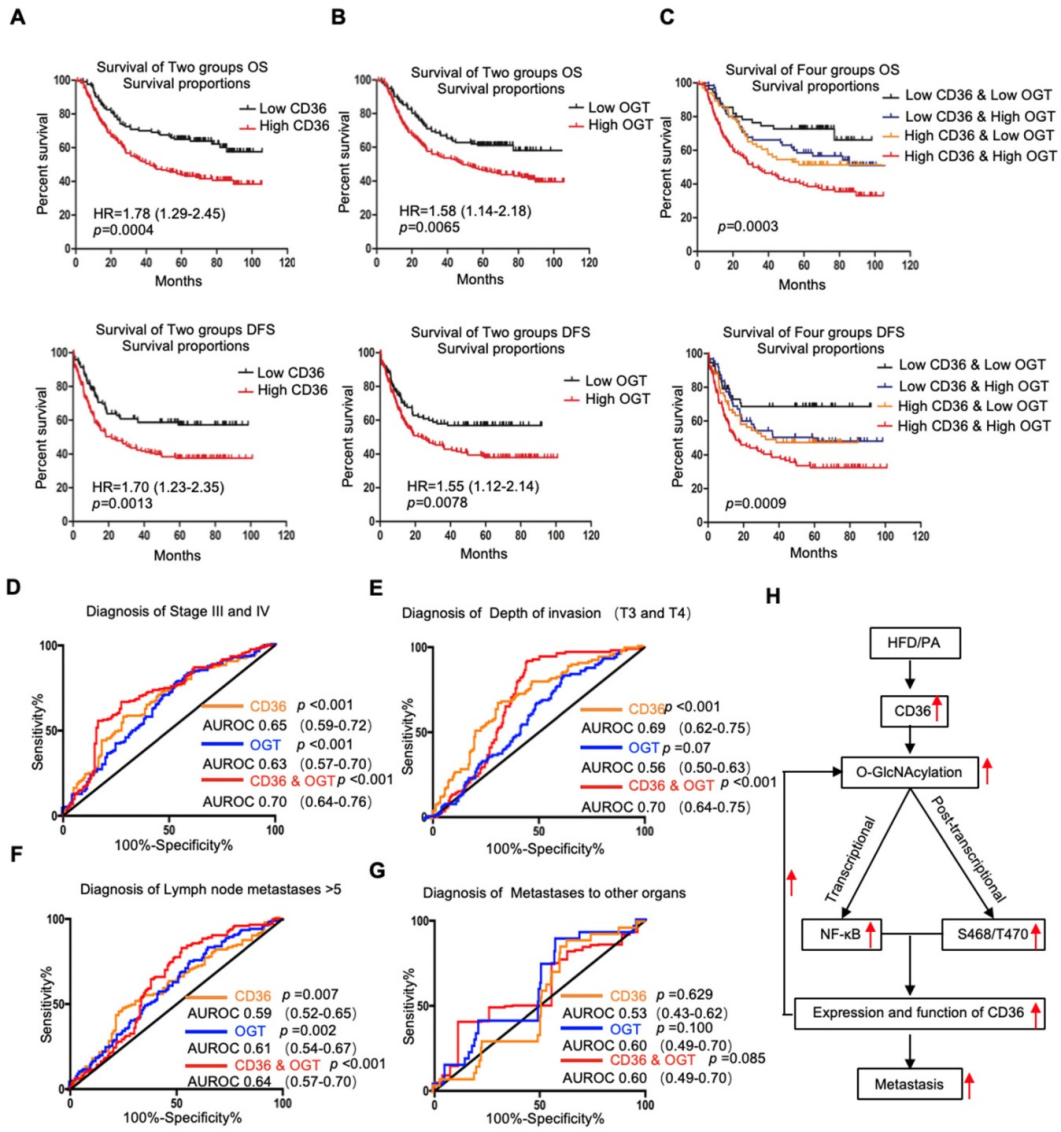
Association of CD36 and OGT expression with the prognosis of GC patients. (A-C) Kaplan-Meier curve depicting the OS and progression-free survival of a publicly available cohort of 300 GC patients (GSE62254). High and low expression of CD36 or OGT were defined by patients whose tumors expressed CD36 or OGT at levels higher and lower than the median, respectively. (D-G) Area under the receiver-operating characteristic (AUROC) curves of CD36 or OGT or both were used to diagnose the indicated patients in all 300 GC patients from GSE62254. (H) Schematic model of Fatty acid-induced CD36 expression via O-GlcNAcylation drives gastric cancer metastasis.

Because OGT is the only known enzyme that catalyzes O-GlcNAcylation in mammals 32 and because the content of OGT often reflects the level of O-GlcNAcylation in cells, we further examined the levels of CD36 and OGT mRNA in a publicly available cohort of 300 GC patients (GSE62254) 33, dividing the patients into groups based on the high or low expression of CD36 or OGT relative to the respective median expression level. We observed that the level of CD36 in GC tissues was significantly associated with age, Lauren type, pStage, depth of invasion and number of positive lymph nodes but was not related to gender, distant metastasis or lymph node metastasis. GC patients with deep tumor invasion (T3 and T4), high pStage (stages III and IV) and more positive lymph nodes (>5) had significantly higher expression of CD36 than those with superficial tumor invasion (T1 and T2), low TNM stage (stages I and II) and fewer positive lymph nodes (≤5) (Table 1). Overall, patients with diffuse-type GC had a higher level of CD36 expression than those with other GC types. Similarly, the level of OGT in GC was significantly associated with Lauren type, pStage, lymph node metastasis and the number of positive lymph nodes but was not related to gender, distant metastasis or depth of invasion. Patients with a higher pStage (stages III and IV), more lymph node metastasis (>5) and a higher N stage (N 2 and N 3) had significantly higher OGT expression than those with a low pStage (stages I and II), less lymph node metastasis (≤5) and a lower N stage (N 0 and N 1) (Table 1). Moreover, patients with diffuse-type GC had a higher level of OGT expression than the other GC types. In addition, a Kaplan-Meier analysis showed that patients with higher levels of CD36 or OGT mRNA had the shortest overall survival (OS) or disease-free survival (DFS) times (Figure 7A and 7B).

Next, we assessed whether the GC prognosis prediction was more accurate using the combined expression of CD36 and OGT rather than the expression of each factor alone. Patients were divided into 4 groups: group 1, low expression of CD36 and low expression of OGT; group 2, low expression of CD36 and high expression of OGT; group 3, high expression of CD36 and low expression of OGT; and group 4, high expression of CD36 and high expression of OGT. Kaplan-Meier analysis showed significantly distinct OS and DFS patterns among the four subgroups. GC patients with high levels of CD36 and OGT had the poorest prognosis. In both the high- and low-OGT-level tumors, patients with tumors exhibiting low CD36 expression had better OS and DFS survival times than patients with tumors exhibiting high CD36 expression, whereas the survival of patients with low CD36/high OGT and high CD36/low OGT was not significantly different (Figure 7C).

To further evaluate the potential utility of using the levels of CD36 and OGT in cancer tissues as an index to evaluate the metastatic potential of GC, the area under the receiver-operating characteristic (AUROC) curve was calculated. We observed that both the CD36 and OGT levels could patients with a higher pStage (stages III and IV) (Figure 7D), deeper tumor invasion (T3 and T4) (Figure 7E) and more positive lymph nodes (>5) (Figure 7F), while the combination of the CD36 and OGT levels showed a high overall accuracy. However, perhaps because there were so few patients with distant metastases, neither OGT nor CD36 levels, or even a combination of the two, could distinguish the patients with distant metastases from all 300 patients (Figure 7G).

## Discussion

In this study, we demonstrated that CD36 is frequently upregulated in GC patients and is positively associated with poor survival and metastasis. CD36 is a cell surface protein that takes up lipids from the extracellular environment and is considered to be a regulator of lipid metabolism 34. Fat is an important energy-supplying substance, and large amounts of fat uptake by tumor cells can provide sufficient energy for distant metastasis. Importantly, studies have shown that CD36 can serve as an effective marker to isolate tumor cells with high metastasis-initiating potential from multiple cell lines and plays a significant role in cancer development through different mechanisms 35-37. Evidence from anti-CD36 neutralizing antibodies, as well as the clinical significance of CD36 in GC patients, suggests that CD36 may behave as an oncogenic factor that contributes to the development and progression of GC.

The molecular mechanisms by which CD36 exerts its broad range of functions in GC metastasis were investigated in this study. Altered lipid metabolism is one of the most important hallmarks of cancer metastasis initiation 38, and CD36 was previously identified as the dominant regulator that may link the bioenergetic pathway to oncogenic signaling through regulatory factors involved in lipid metabolic reprogramming in cancer cells^12^, ^37^. Therefore, it is reasonable to speculate that the upregulation of CD36 associated with metabolic abnormalities may be linked to the cancer cell progression of GC. To investigate whether CD36 plays an oncogenic role by causing abnormal lipid metabolism in GC, we used a xenograft model and observed that mice fed a HFD developed more and larger metastatic nodules in the lungs, which was accompanied by an increase in the percentage of CD36+ cells in lung metastatic lesions (Figure 3A and 3C). These results indicated that CD36 may promote GC metastasis through abnormal lipid metabolism in GC. In agreement with this result, GC cell line treatment with PA led to an increase in both the mRNA and protein levels of CD36 and promoted the expression of CD36 in the membrane (Figure 3D-3F). These findings suggest that the oncogenic effect of CD36 in GC is mediated at least in part by inducing abnormal lipid metabolism.

Recent evidence has suggested that posttranscriptional and transcriptional mechanisms regulated by the HBP act as major players in cancer metabolism 39. As a key nutrient sensor, the HBP has been implicated in lipid metabolism and is closely associated with the transduction of multiple signaling pathways 15, 29-31. We therefore hypothesized that dietary lipids induce CD36 expression and promote GC metastasis by activating the HBP OGT is the only known enzyme to catalyzes O-GlcNAcylation in mammals, and the OGT content often reflects the level of O-GlcNAcylation in cells 32. We observed that the depletion of OGT significantly suppressed PA-induced CD36 expression and that upregulation of O-GlcNAcylation promoted GC cell migration and invasion. Importantly, knockout of OGT or CD36 partly blocked the metastasis induced by a HFD in mice (Figure 4E and 4F). In addition, we observed that after 24 h of 0.4 µM PA treatment, the activity of several classical signal transduction pathways in the SGC 7901 cells changed significantly (Figure S4A and S4B). Cluster analysis revealed that knocking out OGT or CD36 largely reversed the PA-induced changes (Figure S4C). Taken together, these results showed that PA or a HFD specifically promotes the metastatic potential of GC cells, which is partly dependent on CD36 and OGT.

The underlying mechanism by which O-GlcNAcylation promotes CD36 production can be attributed to enhanced transcription of CD36. Elevated O-GlcNAcylation is known to activate several signal transduction pathways in cancer. Our results showed that upregulation of O-GlcNAcylation by TMG treatment significantly increased the levels of CD36 mRNA in cells, whereas the downregulation of O-GlcNAcylation caused by knocking out OGT reduced the expression of CD36 mRNA, indicating that O-GlcNAcylation promotes CD36 transcription. In this study, we observed that O-GlcNAcylation-activated NF-κB accelerated CD36 transcription, suggesting that the tumor-promoting role of CD36 is at least partly dependent on O-GlcNAcylation-mediated NF-κB activation. In addition, O-GlcNAcylation, as an important posttranslational modification, is involved in the regulation of several proteins by directly modifying target proteins, such as p53 40, EZH2 41 and snail-1 42. Therefore, we hypothesized that O-GlcNAcylation may directly modify the CD36 protein. Indeed, O-GlcNAcylation was reported to likely be important for CD36 membrane recruitment and plays a role in LCFA use in the heart 21. In addition, the C-terminus of CD36 is necessary for its plasma membrane localization and function in LCFA uptake 43. Our data revealed that O-GlcNAcylation at S468 and T470, which are located in the C-terminus of CD36, is a positive regulator of CD36 FA uptake activity. We believe that O-GlcNAcylation at S468 and T470 may promote FA uptake by facilitating the plasma membrane localization of CD36, a possibility that requires further investigation. Moreover, there is a close interplay between O-GlcNAcylation and ubiquitination in the control of protein degradation 44, and it is well documented that CD36 is degraded through the ubiquitination pathway 45, 46. Therefore, we suspect that O-GlcNAcylation of CD36 inhibits its degradation by inhibiting its ubiquitination. Indeed, we observed that O-GlcNAcylation enhanced CD36 protein stability by prolonging the half-life of its degradation (data not shown). Although the level of CD36 O-GlcNAcylation decreased after mutating the S468 and T470 residues, further mass spectrometry verification is still needed. Because changes in the primary structure of proteins have a significant impact on the spatial structure, it is possible that CD36 has only one O-GlcNAcylation site, S468 or T470. The S468 and T470 residues of CD36 are extremely close in proximity, and a mutation at S468 or T470 may affect the spatial structure of the adjacent residue and decrease the O-GlcNAcylation of CD36. However, the identification of O-GlcNAc sites by mass spectrometry is difficult, especially for CD36, owing to its high degree of N-linked glycosylation.

## Conclusion

In summary, as shown in Figure 7H, the results of this study demonstrated that high fat uptake by GC cells can provide sufficient energy for metastasis while increasing the level of O-GlcNAcylation. Increased O-GlcNAcylation levels promote the transcription of CD36 by activating the NF-κB pathway, and CD36 can be directly modified by O-GlcNAc, promoting the expression and function of CD36. Furthermore, upregulation of CD36, the key molecule for FA uptake, leads to increased fat uptake by GC cells, forming a vicious cycle that promotes GC metastasis. Thus, our results showed that CD36 may serve as a potential therapeutic target in metastatic GC.

## Supplementary Material

Supplementary materials and methods, figures and tables.

## Figures and Tables

**Table 1:** Association of CD36 or OGT level with clinicopathological parameters of patients with gastric cancer cancer.

Characteristics	Total	CD36		*p* value	OGT		*p* value
		Low	High		Low	High	
Gender							
Female	101	46	55	0.272	48	53	0.541
Male	199	104	95		102	97	
Age (years)							
≤60	117	43	74	<0.001	54	63	0.287
>60	183	107	76		96	87	
Lauren							
Diffuse	134	44	90	<0.001	47	87	<0.001
Intestinal	146	95	51		95	51	
Mixed	17	9	8		6	11	
Indeterminate	3	2	1		2	1	
pStage							
I\II	128	79	49	<0.001	77	51	0.002
III\IV	172	71	101		73	99	
Depth of invasion							
T 1\2	188	115	73	<0.001	99	89	0.233
T 3\4	112	35	77		51	61	
Lymph node metastasis							
N 0\1	169	92	77	0.081	98	71	0.002
N 2\3	131	58	73		52	79	
Metastases to other organs							
M 0	273	139	134	0.313	141	132	0.070
M 1	27	11	16		9	18	
Number of positive lymph nodes							
≤5	153	85	68	0.049	88	65	0.008
>5	147	65	82		62	85	
