# Supplementary material for: Fatty acid-induced CD36 expression via O-GlcNAcylation drives gastric cancer metastasis
Source: Theranostics. 2019 Jul 9;9(18):5359–73. doi: 10.7150/thno.34024 (PMC6691574; doi:10.7150/thno.34024)
Supplement: Supplementary file 1 — Supplementary materials and methods, figures and tables. [file thnov09p5359s1.pdf]

Supporting Information

Fatty acid-induced CD36 expression via O-GlcNAcylation drives gastric cancer metastasis

Mingzuo Jiang[1#], Nan Wu[1,3#], Bing Xu[2#], Yi Chu[1#], Xiaowei Li[4], Song Su[1], Di Chen[1], Wenjiao Li[1], Yanting Shi[1], Xiaoliang Gao[1], Haohao Zhang[1], Zhao Zhang[5], Wei Du[5], Yongzhan Nie[1], Jie Liang[1*], Daiming Fan[1*]*

1, State key Laboratory of Cancer Biology, National Clinical Research Center for Digestive Diseases and Xijing Hospital of Digestive Diseases, Air Force Military Medical University, 710032, Xi'an, China.

2, Department of Gastroenterology, Second Affiliated Hospital of Xi'an Jiaotong University, 710032, Xi'an, China

3, Lab of Tissue Engineering, Faculty of Life Science, Northwest University, 710032, Xi'an, China

4, Department of Gastroenterology, Navy General Hospital, 100048, Beijing, China.

5, The School of Basic Medicine, Air Force Military Medical University, 710032, Xi'an, China.

[#] *These authors contributed equally: Mingzuo Jiang, Bing Xu, Yi Chu, Nan Wu.*

[*] *Corresponding authors: Corresponding to Prof. Daiming Fan, daimingfan@fmmu.edu.cn or Prof. Liang, liangjie@fmmu.edu.cn or Prof. Yongzhan Nie, yongznie@fmmu.edu.cn*

State key Laboratory of Cancer Biology, National Clinical Research Center for Digestive Diseases and Xijing Hospital of Digestive Diseases, Air Force Military Medical University, Xi'an, China.

Keywords: fatty acid, gastric cancer, CD36, O-GlcNAcylation, metastasis

Supplementary Materials and Methods:

Immunoblotting: Cells were lysed in RIPA lysis buffer (Beyotime Biotechnology, P0013B, Shanghai, China) supplemented with a protease inhibitor cocktail (Calbiochem, 539134, San Diego, USA), a phosphatase inhibitor cocktail (Calbiochem, 524627, San Diego, USA) and 10 μmol TMG (Calbiochem, 110165CBC, San Diego, USA). After heating for 5 min at 95°C with 5× SDS-PAGE loading buffer (Beyotime Biotechnology, P0015, Shanghai, China) and electrophoresis, the lysed proteins were transferred to a polyvinylidene difluoride membrane (Merck Millipore, ISEQ00010, Darmstadt, Germany). Then, the membranes were incubated with the indicated antibodies, and the signals were detected in a Bio-Rad ChemiDoc XRS imaging system. The ratio of the gray value of the target protein to that of β-actin represented the relative amount of protein.

Table 1: The list of primary antibodies used

Target	Usage	Source	Catalog number	Dilution
O-GlcNAc	WB	Abcam	Ab2739	1:1000
CD36	WB	R&D	MAB19554	1:800
OGT	WB	CST	5368	1:1000
Flag	WB	Sigma-Aldrich	SAB4200071	1:2000
Ubiquitin	WB	Abcam	Ab134953	1:1000
p65	WB	CST	8242	1:1000
Phospho-p65	WB	CST	3033	1:1000
β-actin	WB	Sigma-Aldrich	A2228	1:5000

abbreviations: WB, western blotting assay; CST, Cell Signaling Technology

Quantitative real-time polymerase chain reaction (qRT-PCR): RNA was extracted from samples using a TaKaRa MiniBEST Universal RNA Extraction kit (TaKaRa, 9767, Tokyo, Japan), and only samples with an OD260/OD280 ratio of 1.8 to 2.0 were used for subsequent analyses. cDNA was obtained by reverse transcription according to the manufacturer's

instructions (TaKaRa, RR036A, Tokyo, Japan). The detection of mRNA levels was performed using a real-time fluorescence quantitative PCR instrument with CFX96 software (Bio-Rad, CA, USA) and SYBR Green Master Mix (Qiagen, 204143, Dusseldorf, Germany). A melting curve analysis was performed, and the relative mRNA levels were normalized to that of β-actin.

Table 2: The list and sequences of primers used for qRT-PCR

Primer	Sequence (5'-3')
OGT-Forward	AGAAGGGCAGTGTTGCTGAAG
OGT-Reverse	TGATATTGGCTAGGTTATTCAGAGAGTCT
CD36-Forward	AAAGTCACTGCGACATGATTAATGG
CD36-Reverse	AACGTCGGATTCAAATACAGCATAG

Cell migration and invasion assay: Cell migration and invasion were assayed using Transwell chambers (6.5 mm; Corning, 3422, NY, USA) with 8-μm pore membranes, with the upper face of the membrane was covered with 60 μL of Matrigel (1 mg/ml) (BD Biosciences, NJ, USA) for cell invasion assays. The supernatant was collected after incubating NIH-3T3 cells in serum-free DMEM medium for 48 h. The lower chamber was filled with 800 μL of the above supernatant with 10% FBS. The cells (2×10^4 cells/well) were suspended in 200 μL of medium containing either 0.1mM PA, 5 μM TMG or its control and added to the upper chamber. After 48 h, the crystal violet-stained cells on the undersurface of the polycarbonate membranes were visually enumerated in five random fields at 100× magnification.

Three-dimensional spheroid BME cell invasion assay: 3D spheroid BME cell invasion assays were performed using a Cultrex® 96-well 3D Spheroid BME Cell Invasion Assay kit (Sigma-Aldrich, 3500-096-K, St. Louis, USA) according to the manufacturer's instructions. Briefly, 3000 cells were resuspended in 50 μL of 1× Spheroid Formation ECM and added to a

96-well Spheroid Formation Plate. Then, the plate was centrifuged at 200 × *g* for 3 min at room temperature and incubated at 37°C in an incubator for 72 h to promote spheroid formation. Next, 50 μL of Invasion Matrix per well was added with the plate on ice, followed by centrifugation at 300 × *g* at 4°C for 5 min. The plate was transferred to a 37°C incubator for 1 h to promote gel formation on the Invasion Matrix, and 100 μL of warm cell culture medium (with or without the indicated compounds at a 2× concentration) was added per well after 1 h. The plate was incubated at 37°C in an incubator for 7 days, and the spheroid in each well was photographed every 24 h using a 4× objective. The images were analyzed using ImageJ, and the changes in the area of the invasive structure were measured to determine the extent of the 3D BME cell invasion in each sample.

FACS analysis: Cells (5×10^5) were incubated for 30 min on ice in the dark with an APC-conjugated anti-human CD36 antibody (Biolegend, 336208, San Diego, USA) or an APC-conjugated mouse IgG2a, k Isotype control (FC) (Biolegend, 400222, San Diego, USA) antibody at a 1:100 dilution in 100 μL of cell staining buffer (Biolegend, 420201, San Diego, USA). Subsequently, the cells were washed twice with cell staining buffer followed by centrifugation at 350 × *g* for 5 min. The cell pellet was resuspended in 0.5 ml of cell staining buffer, and 10 μL of 7-AAD viability Staining Solution (Biolegend, 420403, San Diego, USA) was added, which was followed by an incubation on ice for 5 min in the dark to exclude dead cells. Then, the samples were analyzed with a flow cytometer.

Supplementary figures and figures legends:

Figure S1:

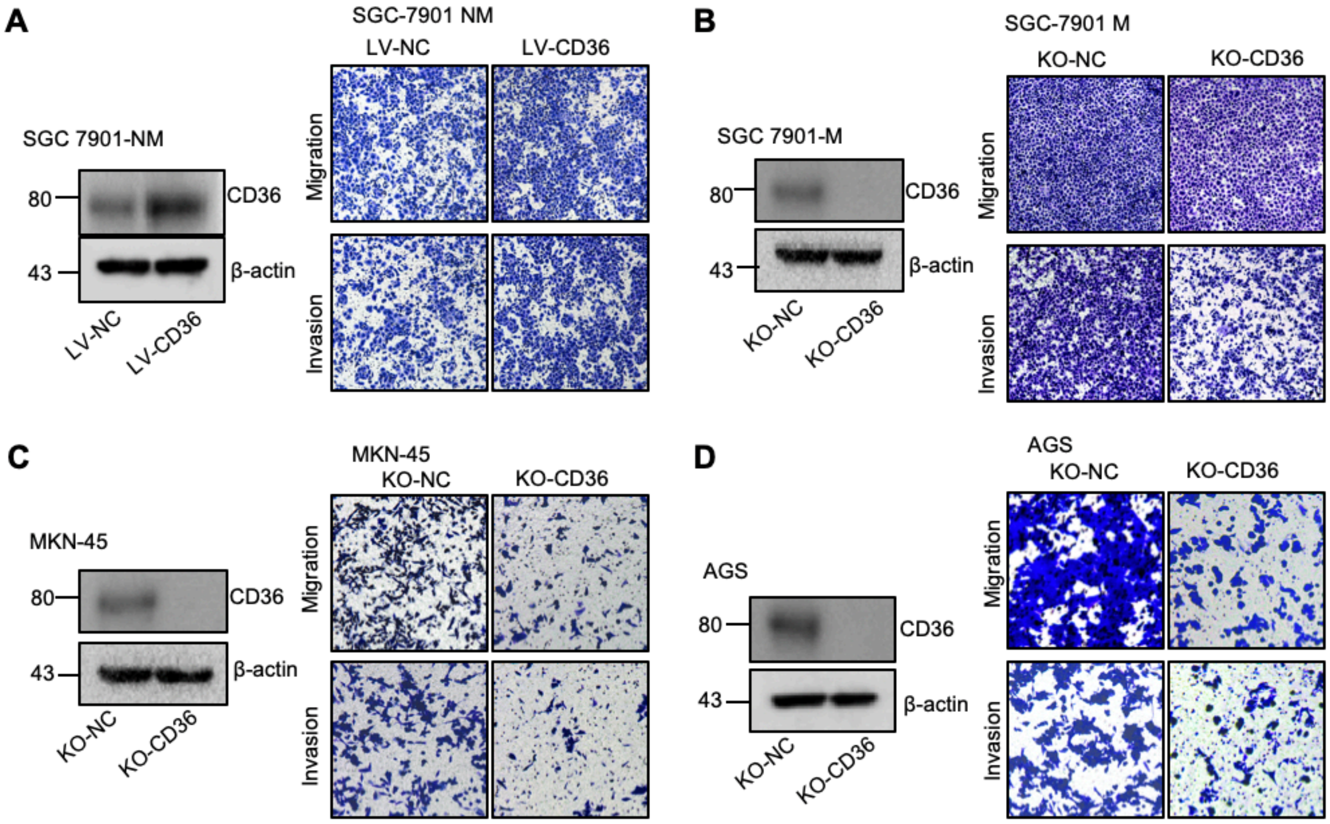

Figure S1 : (A) Transwell migration and invasion assay of SGC 7901-NM cells with CD36 overexpreesion (LV-CD36) or corresponding control cells (LV-NC). The overexpression of CD36 was confirmed by Western Blotting assay. (B-D) Transwell migration and invasion assay of SGC 7901-M (B), MKN-45 (C) and AGS (D) cells with CD36 knockout (KO-CD36) or corresponding control cells (KO-NC). The knockout of CD36 was confirmed by Western Blotting assay.

Figure S2:

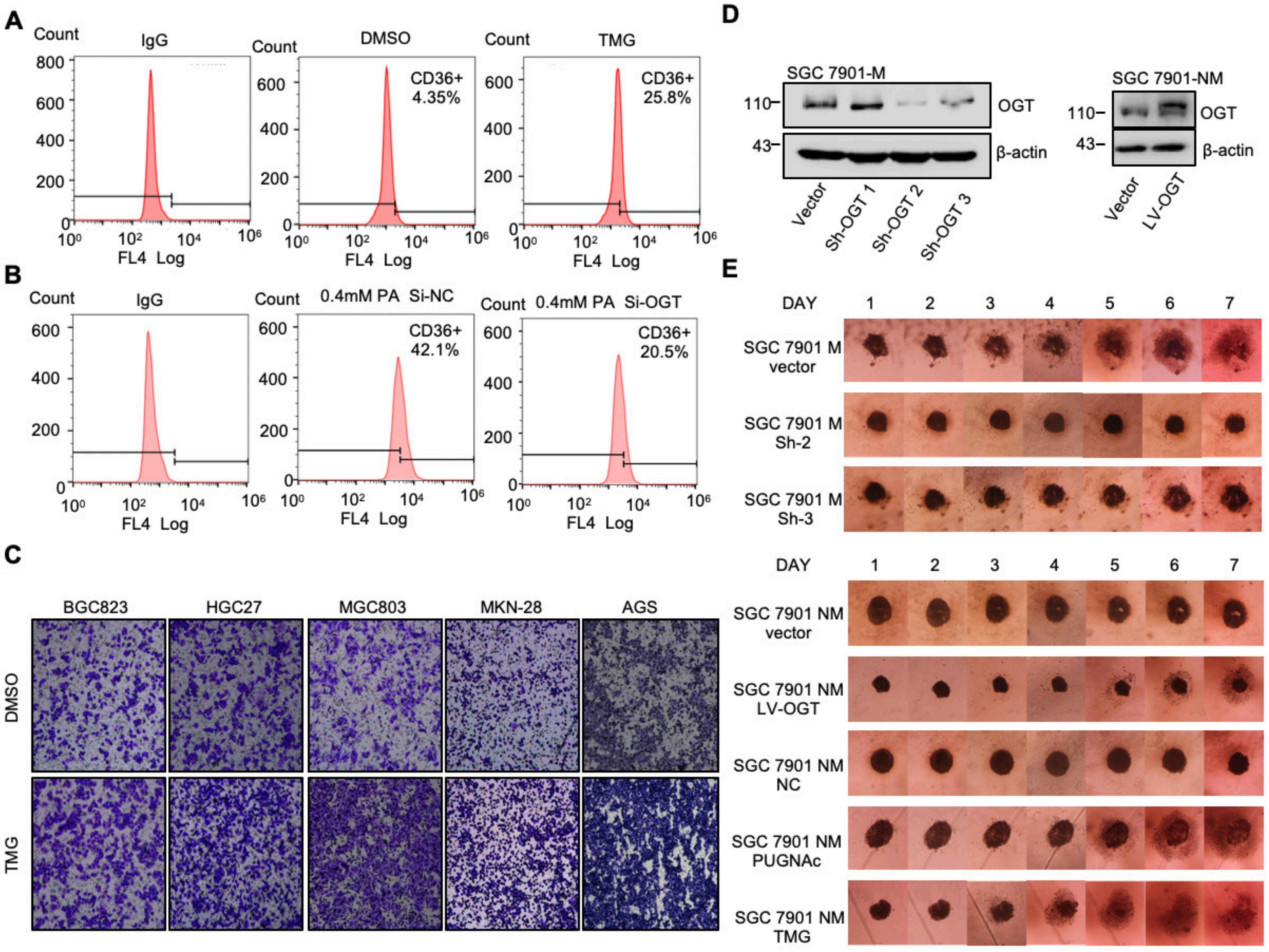

Figure S2: (A) Flow cytometry analysis of CD36 levels in SGC 7901 cells after a 24-hour treatment with TMG (10 μM) or isometric DMSO. K isotype IgG was used as a negative control. (B) Flow cytometry analysis of CD36 levels of SGC 7901 cells with or without OGT silenced by siRNA after treatment with 0.4 μM of PA or isometric control solvent for 24 h. (C) Transwell migration assay of indicated GC cells after10 μM TMG or isometric DMSO treatment for 24 h. (D) SGC 7901-M cells were transfected with LV-OGT-shRNA (Sh-1, Sh-2, and Sh-3) or the control vector, and SGC 7901-NM cells were transfected with lentiviral vector encoding OGT (LV-OGT) or negative control vector (LV-NC). The OGT expression levels were determined by western blotting. (E) 3D spheroid BME cell invasion assay of the indicated

cells or SGC 7901-NM cells with the indicated treatment. Photographs of all the spheroids in each well were taken every 24 h for 7 days using a 4× objective.

Figure S3:

A

O-GlcNAc level	Negative	Weak	Strong	Total
Primary site	11	37	20	68
Adjacent tissue	25	24	4	53
Metastasis	5	25	36	66
Total	41	86	60	187

B

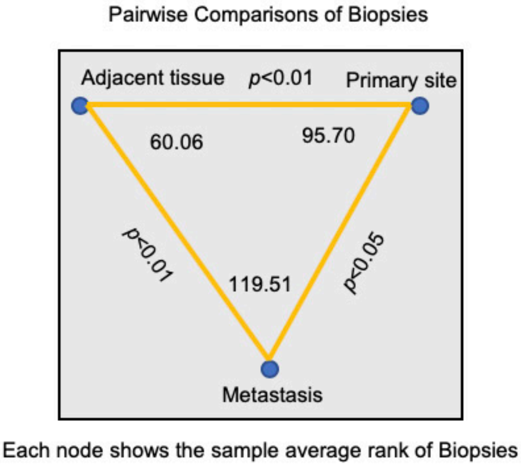

C

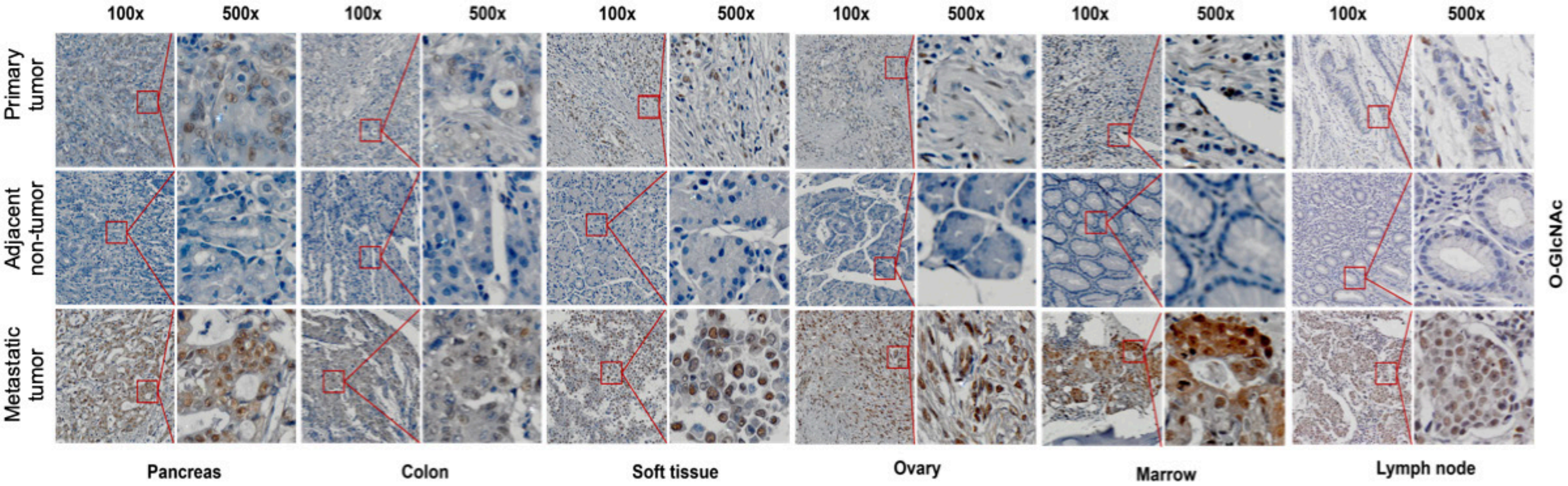

Figure S3: (A) Chi-square analysis of the O-GlcNAcylation level in 53 normal adjacent tissues, 73 primary GC tissues and 61 metastatic GC tissues. The intensity of staining was divided into four grades: negative, weak, moderate and strong. (B) Pairwise Comparisons of O-GlcNAcylation level in adjacent normal tissues, GC tumor tissues, and distant GC metastases. Each node shows the sample average rank of Biopsies and Kruskal-Wallis test was used. (C) Representative images of IHC staining of O-GlcNAcylation and H&E staining in adjacent normal tissues, tumor tissues, and distant metastases (including pancreas, colon, soft tissue, ovary, marrow and lymph node metastases).

Figure S4:

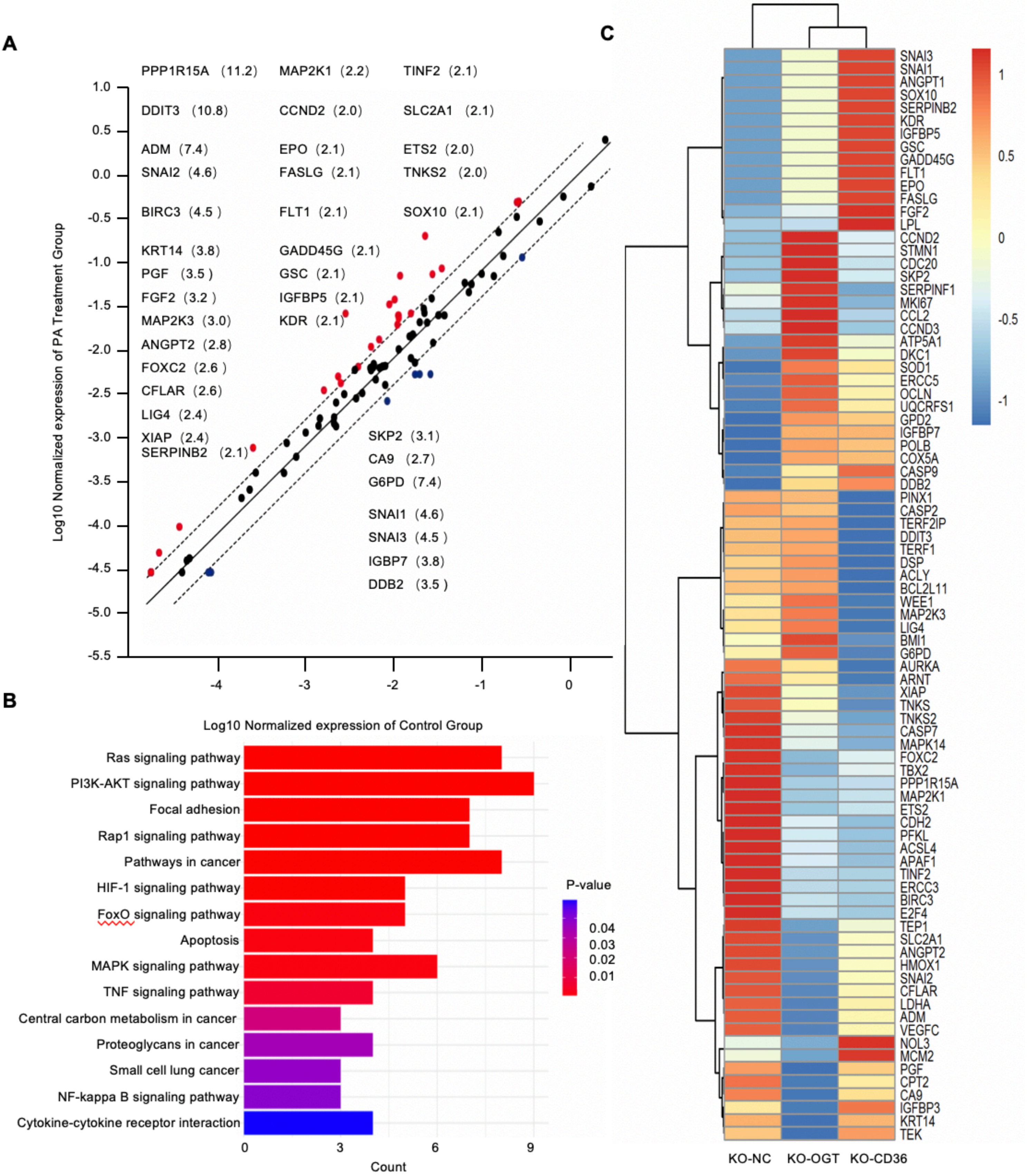

Figure S4: (A) The scatter plot compares the normalized expression of every gene on the array between the PA treatment and control groups (SGC7901 cells were treated with 0.4 μM

PA or isometric control solvent for 24 h) by plotting them against one another to quickly visualize changes in gene expression. The central line indicates unchanged gene expression. The dotted lines indicates the 2-fold regulation threshold. Data points beyond the dotted lines in the upper left and lower right sections meet the 2-fold regulation threshold. (B) GO and pathway enrichment analyses were performed to show the changes in signal transduction pathways between the PA treatment and control groups (SGC7901 cells were treated with 0.4 μM PA or isometric control solvent for 24 h). (C) Cluster analysis revealed that knocking out OGT or CD36 largely reversed the PA-induced changes.

Figure S5:

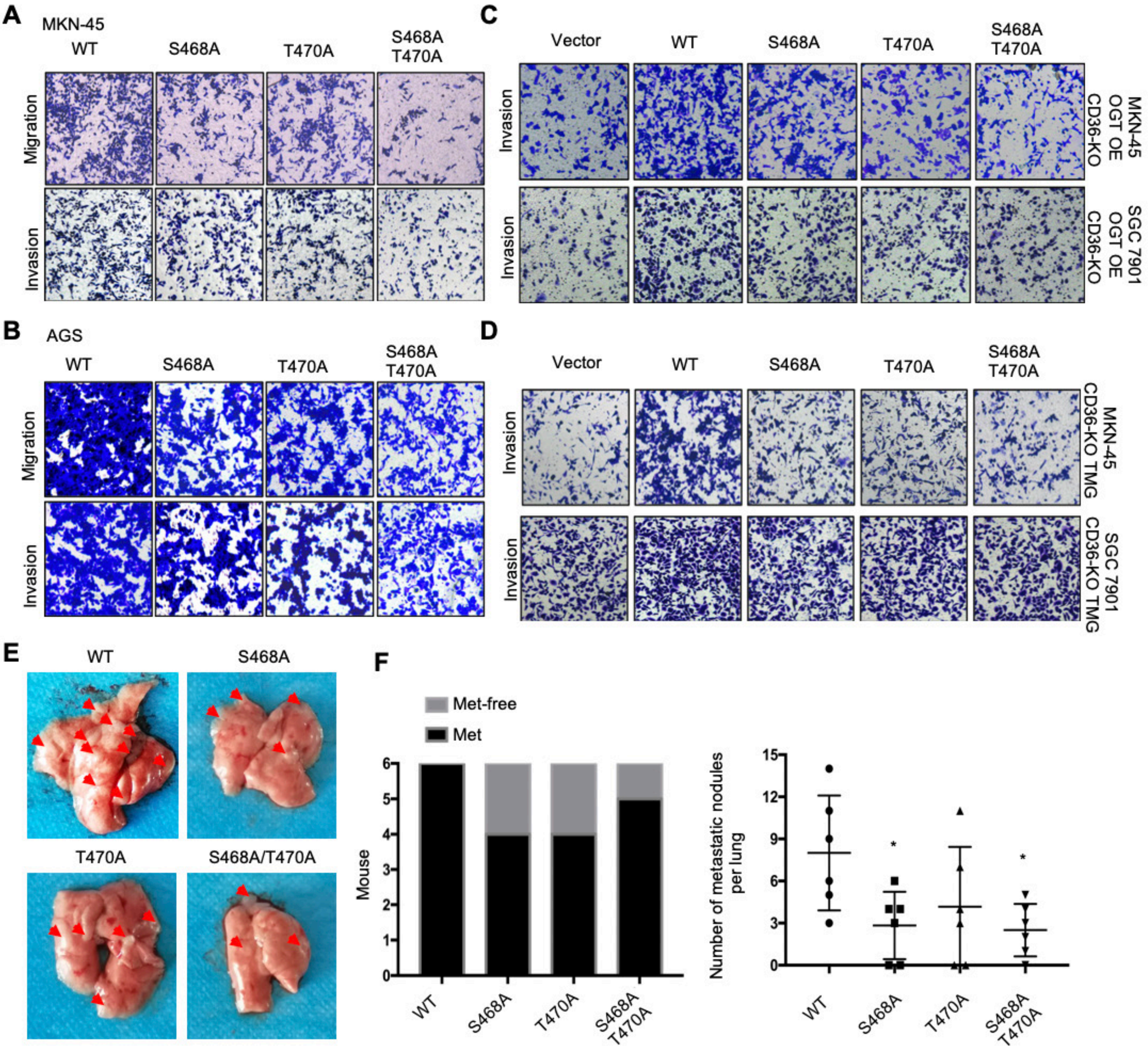

Figure S5：(A, B) Transwell assay of MKN-45 (A) and AGS (B) cells transfected with the indicated lentiviral vector after 0.4 μM PA treatment for 24 h. (C) Transwell invasion assay for indicated cells that re-expression of wild type CD36 or CD36 with S468A, T470A or S468A/T470A mutations by plasmid transfection in SGC 7901 or MKN-45 with OGT overexpression and CD36 knockout. Empty vector plasmid was used as the negative control. (D) Transwell invasion assay for indicated cells after 10 μM TMG treatment that

re-expression of wild type CD36 or CD36 with S468A, T470A or S468A/T470A mutations by plasmid transfection in SGC 7901 or MKN-45 with CD36 knockout. Empty vector plasmid was used as the negative control. (E) Indicated MKN-45 cells were injected into the tail vein of nude mice (n = 6 for each group) fed either a HFD or a normal chow diet. Photos of representative lung tissue samples in each group are shown. (F) Left: The histogram shows the proportion of mice with lung metastasis in each group. "Met" is short for "metastasis," and "Met-free" represents "metastasis-free." Right: Mann Whitney test was used to evaluate the number of metastatic nodes in the lungs of each group.

Figure S6:

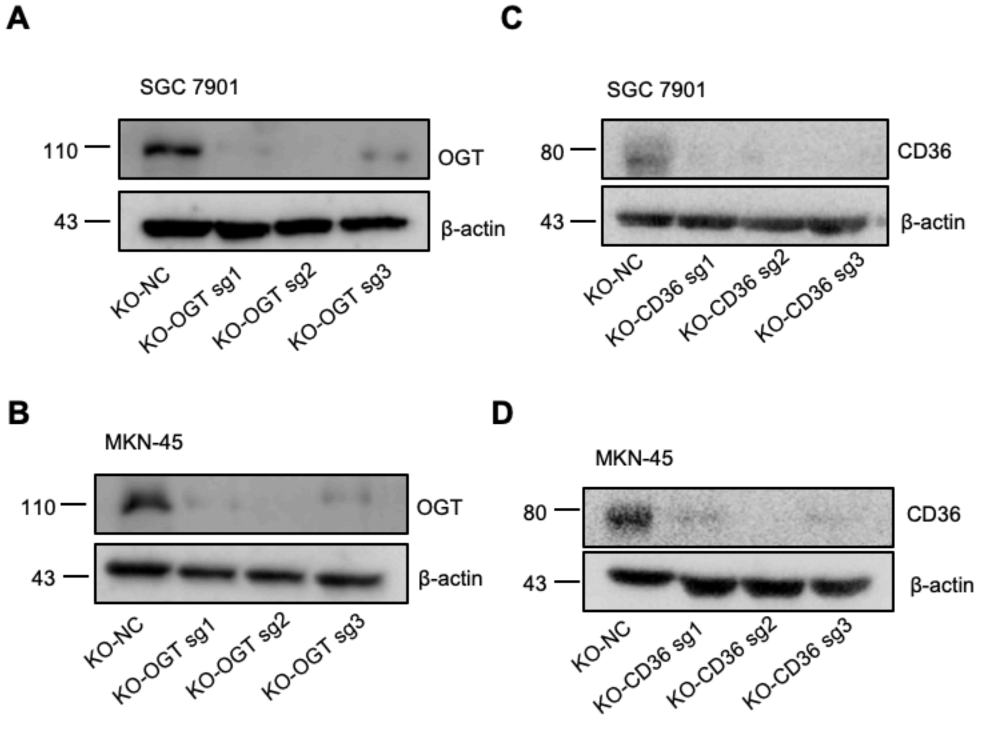

Figure S6: (A, B) Stable OGT knockout SGC 7901 (A) and MKN-45 (B) cells were established via a CRISPR/Cas9-sgRNA system. Puromycin-resistant lentiviral vectors carrying

CRISPR/Cas9 and the indicated OGT-targeted sgRNA (OGT KO-sgRNA1, OGT KO-sgRNA2 and OGT KO-sgRNA3) were transfected into SGC7901 or MKN-45 cells. The levels of OGT in SGC 7901 or MKN-45 cells were assessed by western blotting and β-actin was used as a loading control. (C,D) Stable CD36 knockout SGC 7901 (C) and MKN-45 (D) cells were established via a CRISPR/Cas9-sgRNA system. Puromycin-resistant lentiviral vectors carrying CRISPR/Cas9 and the indicated CD36-targeted sgRNA (CD36 KO-sgRNA1, CD36 KO-sgRNA2 and CD36 KO-sgRNA3) were transfected into SGC7901 or MKN-45 cells. The levels of CD36 in SGC 7901 or MKN-45 cells were assessed by western blotting and β-actin was used as a loading control.
